# Pro- and Anti- Effects of Immunoglobulin A- Producing B Cell in Tumors and Its Triggers

**DOI:** 10.3389/fimmu.2021.765044

**Published:** 2021-11-19

**Authors:** Ziwen Zhong, Ke Nan, Meilin Weng, Ying Yue, Wenchang Zhou, Zhiqiang Wang, Yiwei Chu, Ronghua Liu, Changhong Miao

**Affiliations:** ^1^ Department of Anesthesiology, Zhongshan Hospital, Fudan University, Shanghai, China; ^2^ Department of Anesthesiology, Affiliated Hospital of North Sichuan Medical College, Nanchong, China; ^3^ Department of Immunology, School of Basic Medical Sciences, Fudan University, Shanghai, China; ^4^ Shanghai Fifth People’s Hospital and Key Laboratory of Medical Epigenetics and Metabolism, Institutes of Biomedical Sciences, Fudan University, Shanghai, China

**Keywords:** B cells, plasma cells, immunoglobulin A, tumor immunity, class switching

## Abstract

B cells are well known as key mediators of humoral immune responses *via* the production of antibodies. Immunoglobulin A (IgA) is the most abundantly produced antibody isotype and provides the first line of immune protection at mucosal surfaces. However, IgA has long been a divisive molecule with respect to tumor progression. IgA exerts anti- or pro-tumor effect in different tumor types. In this review, we summarize emerging evidence regarding the production and effects of IgA and IgA^+^ cells in the tumor microenvironment (TME). Moreover, we discuss that the TME cytokines, host diet, microbiome, and metabolites play a pivotal role in controlling the class-switch recombination (CSR) of IgA. The analysis of intratumoral Ig repertoires and determination of metabolites that influence CSR may help establish novel therapeutic targets for the treatment of cancers.

## Introduction

Immunoglobulin A (IgA) is the most abundant antibody class present on mucosal surfaces. It plays a key role at these surfaces, which are continuously exposed to antigens, food, and commensal microorganisms (1). IgA-producing plasma cells (IgA^+^ PCs) are derived from B cells and undergo antibody class-switch recombination (CSR). Emerging evidence shows significant tumor infiltration by B cells. However, the role of B cells and their antibodies in tumors has remained elusive, perhaps due to their phenotype, antibody isotype and production, localization, and the tumor type and tumor microenvironment (TME) (2–5). Multiple lines of evidence now leave no doubt that B cells undergo clonal proliferation, selection for high-affinity antibodies, and isotype switching within tumor-associated tertiary lymphoid structures (TLS) and in less organized structures, ultimately converting into effector/memory B cells and antibody-producing PCs (6–9). However, under certain conditions, tumor-infiltrating B cells, PCs, and their antibodies may exert prominent immunosuppressive effects. Studies have provided well-founded evidence of discrete subsets of immunosuppressive or regulatory B cells (Bregs) in both mice and humans. Their function depends on immunosuppressive factors, such as IL-10, PD-L1, and/or TGF-β (10–13). Moreover, IgA has recently been recognized as a key biomarker of immunosuppressive B cells. However, the effect of IgA^+^ cells and their antibodies on cancer development remains controversial (1, 11, 14–16). In this review, we summarize recent findings related to the production and effect of IgA and IgA^+^ cells in the TME and discuss their potential roles in novel cancer treatment strategies.

## Source and Location of IgA

Approximately 80% of all human PCs are located in organized gut-associated lymphoid tissue (GALT). In GALT, IgA is produced at a constant synthesis rate of 50 mg/kg/day, which is more than the total synthesis rate of other Ig isotypes, indicating that continuous secretion of IgA requires an enormous amount of energy in mammalian species (17). In addition, IgA is present in milk and bronchial secretions (18). Serum IgA is predominantly monomeric in humans and is produced by local PCs of the bone marrow, spleen, and lymph nodes (1). IgA at mucosal surfaces is generally produced by local PCs as dimeric molecules, although small amounts of monomers, trimers, tetramers, or polymers can also be present. Dimeric IgA interacts with the polymeric Ig receptor (pIgR), an antibody transporter expressed on the basolateral surface of intestinal epithelial cells (IECs). A secretory IgA (sIgA) complex is formed after a secretory component (SC) of pIgR binds to IgA through a joining (J) chain. SC is a hydrophilic and highly (N- and O-linked) glycosylated negatively charged molecule that protects sIgA from degradation in luminal secretions (19).

**Figure 1:**
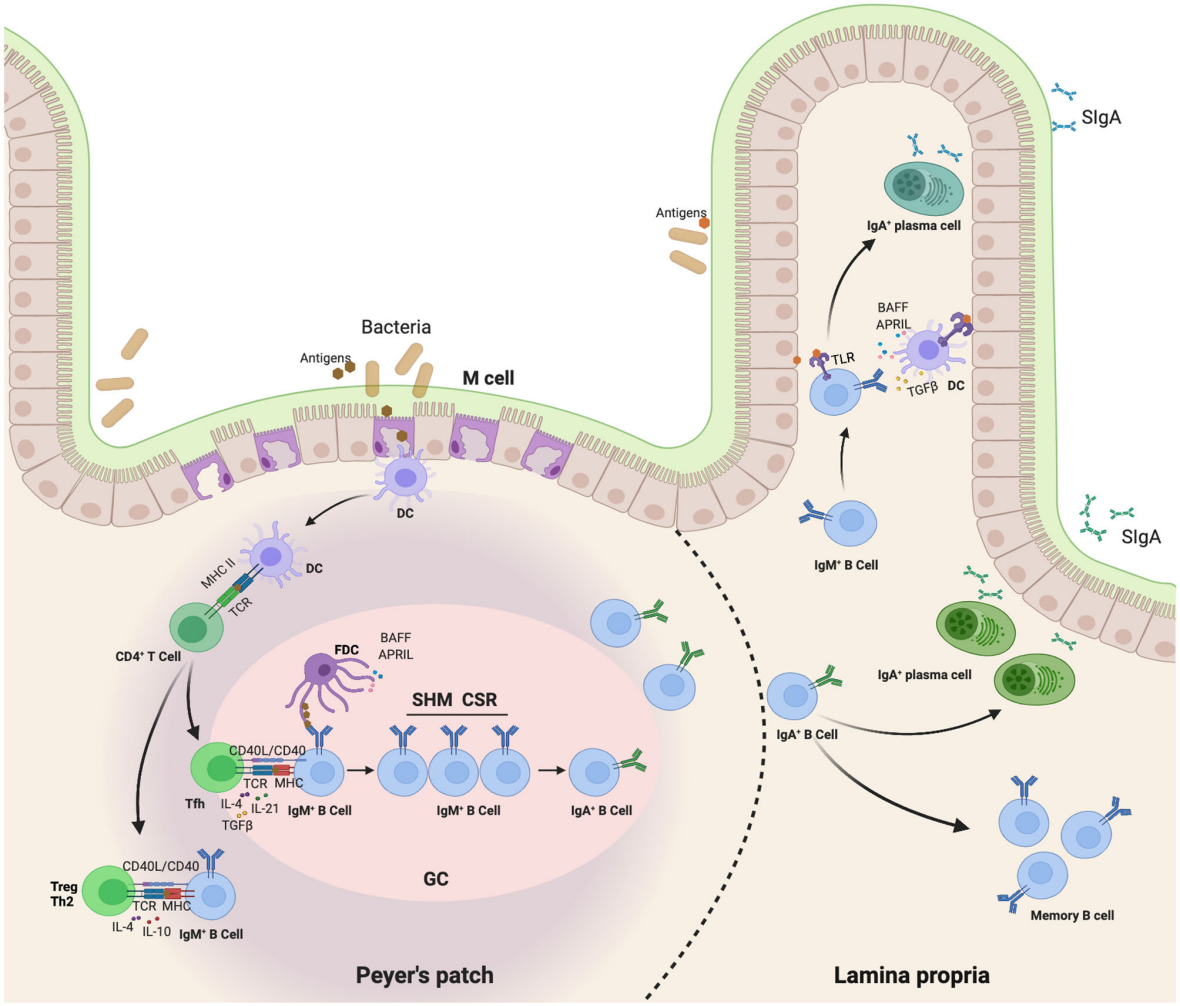
Map of IgA class switching through both TD and TI reaction in the gut. This TD pathway involves a cognate interaction of follicular B cells with antigen-activated CD4^+^ T cells, including TH2 cells, regulatory T (Treg) cells, and T follicular helper (TFH) cells (22, 23). First, Dendritic cells (DCs) in the subepithelial dome (SED) of the PP capture and present antigen to follicular CD4^+^ T cells, thereby inducing them to differentiate into effector T cells releasing IgA-inducing cytokines. These T cells also interact with antigen-specific IgM^+^IgD^+^ naive B cells. Together with follicular dendritic cells (FDCs), this interaction fosters a proliferation, differentiation, somatic hypermutation (SHM) and IgA class-switch recombination (CSR) of B cells in germinal center. Ultimately, TD pathway leads to the emergence of long-lived memory B cells and plasma cells that release high-affinity IgA antibodies in the lamina propria (20). In addition, commensals trigger multiple Toll-like receptors (TLRs) to stimulate FDC, DC and stromal cells release of BAFF, APRIL, TGFβ and other IgA-inducing cytokines, thereby triggering direct IgA CSR in lamina propria IgM^+^ B cells in a T-cell-independent manner (24, 25).

It has been confirmed that various structural compartments differ in the mode of IgA induction. Mucosal antigens initiate IgA production through both T cell-dependent (TD) and T cell-independent (TI) reactions (20–22). Most antigens produce IgA by activating follicular B cells in the germinal center of Peyer’s patches (PPs) and mesenteric lymph nodes (MLNs). This TD pathway involves a cognate interaction between follicular B cells and antigen-activated CD4^+^ T cells, including TH2 cells, regulatory T (Treg) cells, and T follicular helper (TFH) cells (22, 23). Together with follicular dendritic cells (FDCs), this interaction fosters proliferation, differentiation, somatic hypermutation (SHM), and IgA CSR of B cells in the germinal center. Ultimately, this TD pathway leads to the emergence of long-lived memory B cells and PCs that release high-affinity IgA antibodies in the lamina propria (LP) (20). However, TD antibody responses require 5-7 days, which can be too much of a delay to control pathogens. To compensate for this limitation, a TI reaction occurs in PPs, MLNs, isolated lymphoid follicles (ILFs), and LP. Commensals trigger multiple Toll-like receptors (TLRs), which stimulate FDCs, DCs, and stromal cells to release BAFF, APRIL, TGF-β, and other IgA-inducing cytokines, thereby triggering the induction of TI IgA CSR and production in PPs, MLNs, ILFs, and LP (24, 25). Class-switched B cells emerging from these pathways ultimately release both low- and high-affinity IgA antibodies in LP. Moreover, it is likely that the generation of IgA^+^ PCs depends on the nature of IgA and the initial location of B cells. A precise combination of different leukocyte and mesenchymal cell types may also influence the generation of IgA^+^ PCs (23) (**Figure 1**).

## Immunosuppressive B Cells in Tumors Marked With IgA

The tumor-promoting effects of B cells and PCs are generally associated with the presence of heterogeneous cell populations called Bregs and immunosuppressive plasma cells (ISPCs), which may accelerate tumor progression through various mechanisms. Multiple studies have shown that Bregs suppress tumor immunity through immunosuppressive factors, such as IL-10, TGF-β, PD-L1, FASL, IL-35, and Tim1 (11, 14, 26–28). This type of tumor immunity suppression is activated by signals from CD40, TLRs, and B cell receptors (BCRs) (10, 29). Furthermore, Bregs reportedly cause cytotoxic T lymphocyte (CTL) suppression by inducing M2 macrophage polarization (30, 31) and Tregs in squamous carcinomas (SCCs) and pancreatic ductal adenocarcinoma (PDAC) (32).

**Figure 2:**
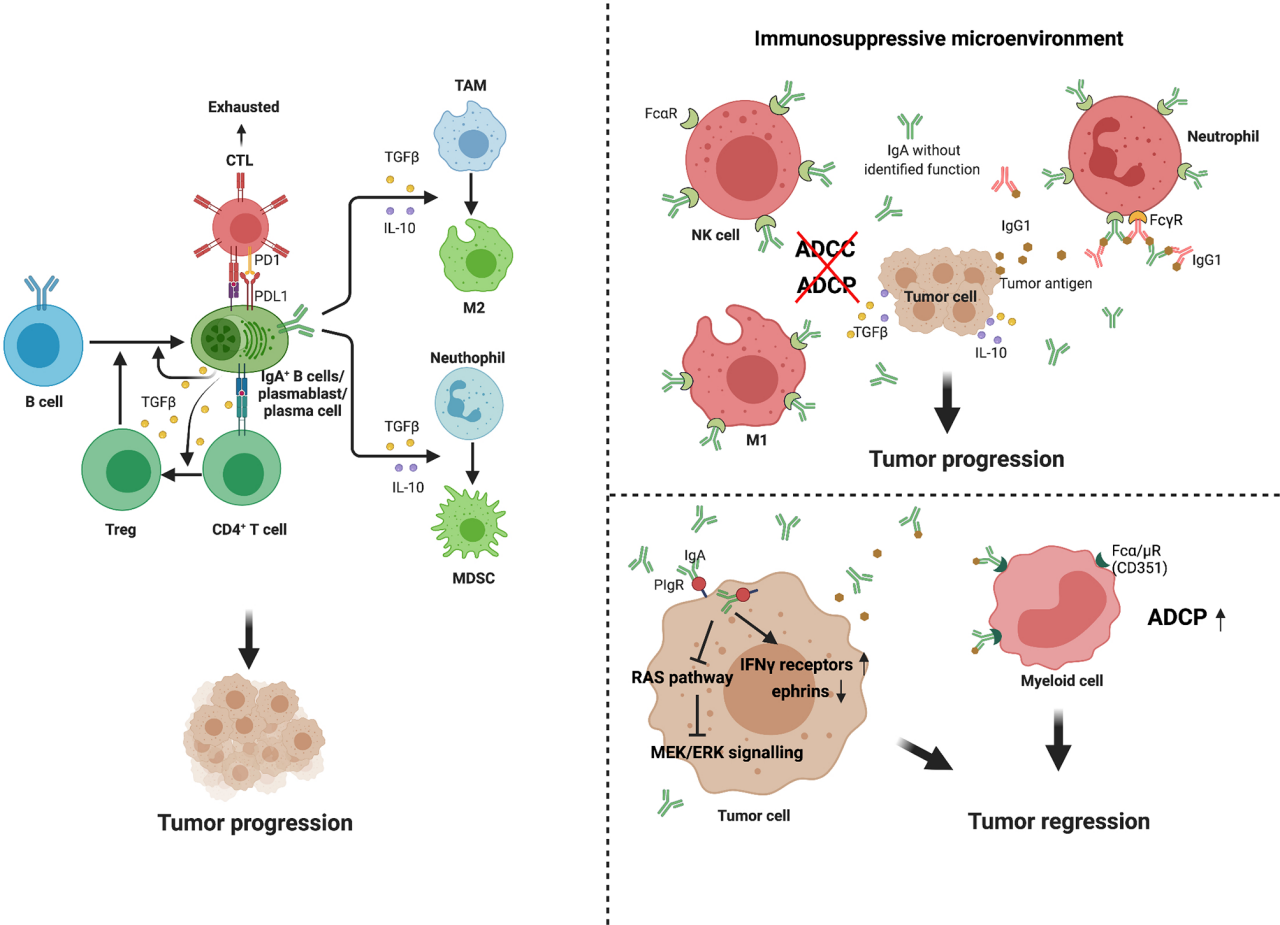
Multiple functions of IgA^+^ cells and IgA in tumor. IgA^+^ B cells and plasma cells may promote tumor progression through several mechanisms. First, they can release high level of PD-L1, IL-10 and TGFβ, which directly induce CD8^+^T cell exhaustion and suppress anti-tumor CTL responses (14, 36, 37). Second, the IgA^+^ cells promote expansion of Treg cell populations, while Treg cells produce TGFβ, which mediates isotype class switch to IgA (9, 38). In addition, these immunosuppressive cytokines secreted by IgA^+^ cells, such as IL-10 and TGFβ, can promote immunosuppressive phenotypes in macrophage and myeloid cells. IgA performs dual effects on tumor immunity. In immunosuppressive environment, due to the binding of IgA without identified function and Fcα receptors (FcαR) expressed on macrophages, natural killer (NK) cells and neutrophils, high levels of production of IgA block ADCC and ADCP (39, 40). Meanwhile, such antibodies can form immune complexes with tumor or non-tumor antigens, promoting chronic inflammation, tissue remodeling (41–43). However, in some specific tumor types, IgA transcytosis induced broad transcriptional changes in inflammatory pathways in tumor cells, contributing to hindering malignant progression. In further support of antibody-dependent cellular phagocytosis, tumor-derived IgA redirection of Fcα/µ^+^(CD351) myeloid cells against extracellular oncogenic drivers, which causes tumor cell death (15).

However, no specific transcriptional marker has been identified that exclusively defines the Bregs phenotype in mouse or humans. Numerous studies have now demonstrated that Bregs mainly suppress inflammatory responses *via* IL-10-depengdent and -independent mechanisms (33). Subsets that are enriched for IL-10-producing B cells include transitional-2 marginal zone precursor (T2-MZP) B cells, peritoneal B-1 B cells, and antibody-producing B cell subsets in mice, as well as antibody-producing and immature B cell subsets in humans. In a recent series of studies, recirculating IgA^+^ regulatory B cells have been shown to suppress inflammation *via* expression of IL-10 (34, 35). Shalapour et al. discovered in 2015 that IgA^+^ PCs suppress anti-tumor immunity. These immunosuppressive B cells are IgA-producing PCs that express PD-L1, IL-10, and Fas-L (11). Most tumoral CD19^+^ B cells in oxaliplatin-treated pancreatic cancer are IgA-positive, and successful eradication of tumors requires removal of IgA^+^ cells (11). In a murine study of hepatocellular carcinoma, IgA^+^ cells expressing high levels of PD-L1, IL-10, and TGF-β were shown to directly repress CD8^+^ T cell proliferation and activation (14). IgA^+^ PCs within prostate tumors induce CD8^+^ T cell exhaustion and suppress anti-tumor CTL responses through PD-L1 and IL-10, either of which can induce anergy or exhaustion (36, 37). In general, IgA^+^ cells promote the expansion of Treg cell populations, whereas Treg cells produce TGF-β, which mediates the isotype class switch to IgA (9, 38). This regulatory loop induces a state of relative immune suppression and may further promote tumor progression in at least some cancer types or subtypes (**Figure 2**).

## Dual Effect of IgA on Tumor Immunity

Although there is more than sufficient evidence indicating that IgA^+^ B cells and PCs may exert prominent immunosuppressive effects, the antitumor effect of IgA^+^ cells has also been reported; this may have resulted from the dual effect of IgA. The expression of IgA by tumor-infiltrating PCs has been linked to poor outcomes in colorectal cancer (CRC) (44) and melanoma (45, 46). Elevated levels of intratumoral IgA have been shown to be associated with poor prognosis and shorter survival in patients with bladder cancer (47). A recent meta-analysis indicated a strong association between IgA CSR and solid cancer diagnosis, and confirmed its immunosuppressive and pro-tumorigenic roles (48). The aforementioned study showed that, compared with healthy individuals, patients with solid malignancies had significantly higher serum IgA levels, which further increased in patients with advanced cancer. However, Biswas et al. demonstrated that tumor-antigen-specific and tumor-antigen-independent IgA responses antagonize the growth of ovarian cancer by governing coordinated tumor cell, T cell, and B cell responses (15). These results suggest that IgA may play various roles in different tumor types or in the tumor immune microenvironment (**Table 1**).

**Table 1:** Mechanism and prognostic impact of IgA in cancers.

Cancer type	Number of patients	Animal models	Methods	Prognostic	Response to therapy	Mechanism		Ref.
						IgA-mediated effects	Beyond IgA production	
Prostate	87	Transplanted tumor models TRAMP transgenic tumor models	IHC, Serum	IgA↓	oxaliplatin↓	N/A	TGFβ receptor signaling↑	(11)
Hepatocellular	598	MUP-µPA, STAM, CCl4, MCD models	IHC, FC, Serum	IgA↓	N/A	N/A	PD-L1↑, IL-10↑	(14)
Ovarian	534	Transplanted tumor models	IHC, TCGA, Single-cell V(D)J (BCR) sequencing	IgA↑	N/A	IgA transcytosis	N/A	(15)
Colorectal	90	AD model	IHC	IgA↓	N/A	N/A	PD-L1↑, IL-10↑, TGFβ↑	(44)
Melanoma	710	N/A	IHC	IgA↓	N/A	Inability to activate the complement cascade, low affinities for activating FcRs、 low potency in triggering ADCC and ADCP	N/A	(45, 46, 49)
Bladder	110	N/A	IHC	IgA↓	N/A	N/A	N/A	(47)
LUAD	442	N/A	TCGA	IgA↓	N/A	N/A	PD-L1↑, IL-10↑	(4)
AML	13	N/A	TCGA, scRNA-seq	IgA↓	N/A	N/A	PD-L1↑, TGFβ↑	(50)
Breast	66	N/A	Serum, IHC	IgA→ IgA-Fc-folate conjugate↑	N/A	ADCC	N/A	(51, 52)
Head and neck	97 226	N/A	Serum	IgA→ IgA↓	N/A	N/A	N/A	(53, 54)
Pancreatic	812073	N/A	Serum, Urine	IgA→	N/A	N/A	N/A	(55)

Prognostic: favorable/mostly favorable ↑ no impact → unfavorable ↓.

### Pro-Tumor Effect of IgA

A systematic review and meta-analysis revealed that a 2-fold increase in the standardized mean difference (SMD) of the IgA level occurred in advanced cancer compared with early carcinoma, further supporting the possibility that serum IgA levels may correlate with immune escape and tumor burden (48). A high intratumoral proportion of the IgA isotype has a close relationship with a negative prognosis in the KRAS-mutant subtype of lung adenocarcinoma (4). TCGA RNA-seq data of human melanoma indicate that high proportions of IgA, IgD, and IgE are related to a poor prognosis (2).

The tumor-promoting mechanism of IgA can be summarized as follows. Firstly, it is a tumor immunosuppressive environment and not a tumor antigen that promotes IgA production. IgA with unfocused specificity is ineffective in mediating antitumor responses, for which it cannot facilitate antigen presentation or mediate antibody-dependent cellular cytotoxicity (ADCC) and phagocytosis (ADCP) of tumor cells (39, 40). On the other hand, such antibodies can form immune complexes with tumor or non-tumor antigens, promoting chronic inflammation and tissue remodeling, and thus directly or indirectly favoring the myeloid-derived suppressor cell phenotype (41–43) (**Figure 2**). Another possible role of mucosal IgA in tumor immunity depends on its dynamic relationship with environmental factors, such as microbiota or diet (56). Tissue-specific immunoregulation may determine the ability of different tumors to exploit the IgA–microbiota axis to facilitate immune escape (56, 57). Furthermore, IgA fails to activate the complement system but mediates the regulatory effects of its main inducer, TGF-β (58). In particular, monomeric IgA exerts inhibitory effects on many immune cell subsets by activating the myeloid-cell-specific type I Fc receptor for IgA (FcαRI; also known as CD89) (59, 60) and inducing IL-10 production (61), as well as by regulating proinflammatory cytokines (62).

### Anti-Tumor Effect of IgA

IgA also plays an anti-tumor role in some specific cancers. Recent studies have demonstrated that tumor-derived IgA abrogates tumor growth through antigen-dependent redirection of Fcα/µ^+^ myeloid cells and antigen-independent, pIgR-mediated transcytosis (15). IgA can bind to polymeric IgA receptors that are universally expressed on ovarian cancer cells. IgA transcytosis induces broad transcriptional changes in inflammatory pathways in tumor cells, including the upregulation of IFN-γ receptors and downregulation of tumor-promoting ephrins. In addition, IgA transcytosis through malignant epithelial cells can antagonize the RAS pathway and sensitize tumor cells to cytolytic killing by T cells, which also helps prevent malignant progression (**Figure 2**). Thus, tumor-antigen-specific and tumor-antigen-independent IgA responses inhibit the growth of ovarian cancer cells by regulating coordinated B cell, T cell, and tumor cell responses.

These results indicate that IgA plays a completely different role in governing ovarian cancer than that in some other types of tumors. The causes may be related to the tumor type and TME. It can be hypothesized that most solid tumor cells do not express polymeric IgA receptors that are universally expressed on ovarian cancer cells, and hence IgA fails to exert anti-tumor effects through transcytosis. Furthermore, some tumor antigens can be specifically recognized by IgA in ovarian cancer. However, in some other tumor types, tumor antigens may not be exposed and thus cannot be recognized on the surface of tumor cells, or do not contain immunogenic peptides suitable for presentation to T cells on major histocompatibility complex class (MHC) molecules, which may lead to failure of recognition by IgA and thus mislead the immune response and favor pro-tumor processes (40, 63). Therefore, the analysis of intratumoral IgA expression and clonality is crucial for evaluating the role of IgA in tumors.

## Tumor-Related IgA Class Switching

Emerging evidence points that a combination of host, environmental, and tumor factors mediates IgA class switching and determines the efficiency of cancer surveillance or promotion (48, 64). At the host level, this implies that increased serum IgA levels are related to older age, male gender, metabolic syndrome, etc. (48, 64, 65). Recent study has shown that environmental factors, including commensal bacteria and diet, and tumor immunosuppressive cytokines, including IL-10 and TGF-β, affect the production of IgA (66–68).

### CSR Triggers in Immunosuppressive TME

In the TME, most tumor-infiltrating TH/TFH cells present a regulatory phenotype, which secretes immunosuppressive cytokines and supports the development of immunosuppressive and anti-inflammatory isotypes (69). Furthermore, activated cancer-associated fibroblasts (CAFs) and tumor-associated macrophages (TAMs) in the TME can produce TGF-β, which represses NK cell and CTL activation, promotes Tregs, and supports IgA CSR (38, 70, 71). More precisely, naïve B cells are recruited into the TME by CAFs *via* CXCL13 and CXCL12 and further exposed to TGF-β, IL-21, IL-33, lymphotoxin β (LTβ), and IL-10, which favors IgM-naïve BCR class switching to IgA (11, 70). Emerging evidence shows that tumors can also induce IgA CSR through a regulatory loop formed by myeloid-derived suppressor cells (MDSCs) and IgA. A recent study showed that CD11b^+^ MDSCs induced the differentiation of B cells into IgA^+^ PCs by secreting IL-10 and TGF-β (72). IgA with unfocused specificity can form immune complexes simultaneously with tumor or non-tumor antigens, promoting immunosuppressive phenotypes in myeloid cells (40).

### CSR Triggers From Host Diet, Microbiome, and Metabolites

**Figure 3:**
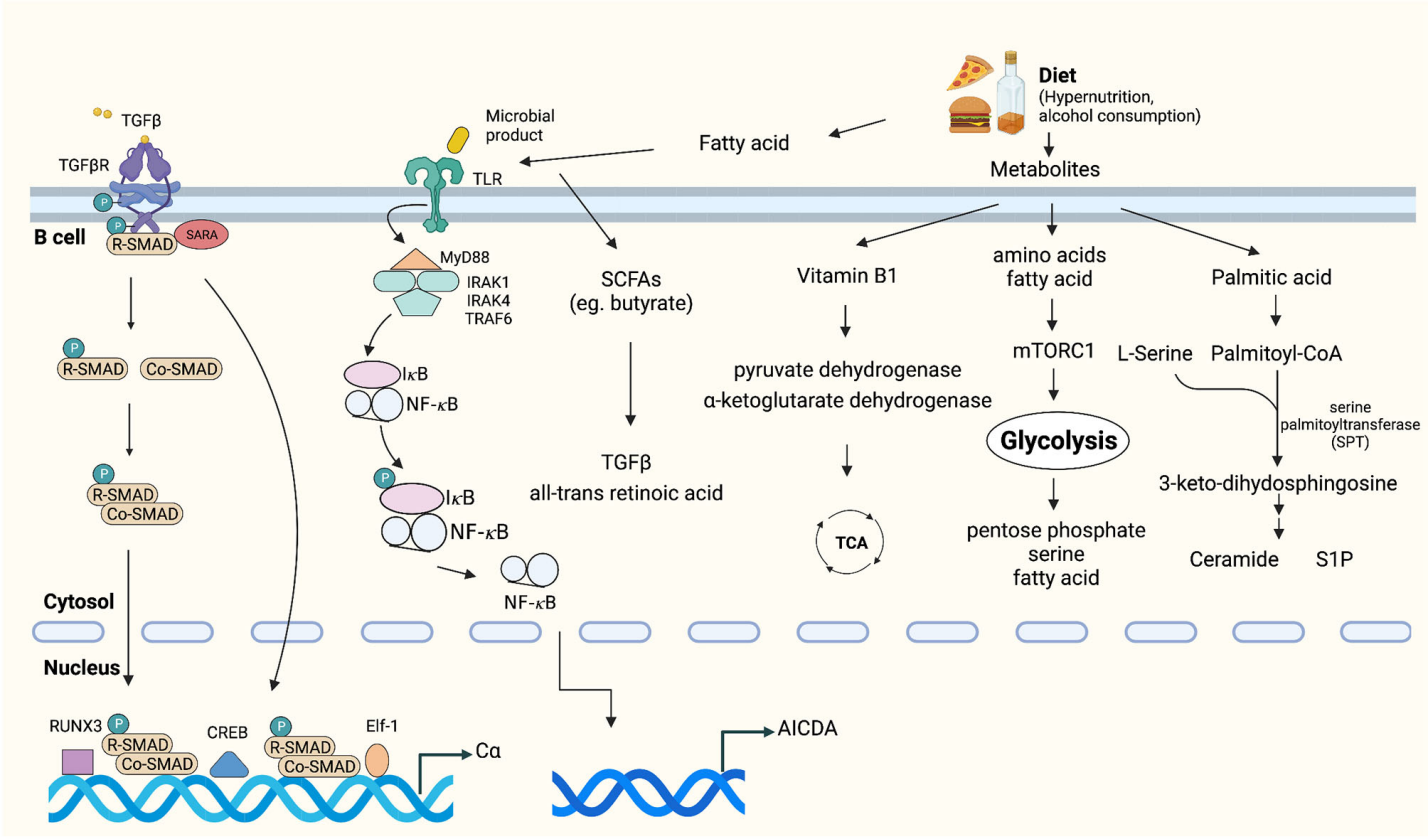
Tumor microenvironmental stimuli that induce IgA class switching. TGFβ is highly expressed in the tumor microenvironment, which activates the constant heavy chain α (Cα) gene promoters (38, 70, 71). Exposure to dietary extracellular metabolites within the immediate microenvironment may play an important role in controlling the production of IgA (73–75, 77). Fatty acid or microbial products can activate TLRs, which promote activation of IκB kinase (IKK) complex, phosphorylation and degradation of IκB [inhibitor of nuclear factor-κB (NF-κB)]. Subsequently, IκB-free NF-κB translocates to the nucleus and initiates CSR by binding to κB motifs on the AID gene promoter (81–83).

Diet is universally considered a mechanism that can affect energy metabolism and immune responses, including intestinal IgA production (73). Among the various dietary components, vitamins are the most important factors for immune responses. For example, vitamin B1 has been confirmed to play a pivotal role in intestinal IgA responses as a cofactor of pyruvate dehydrogenase and α-ketoglutarate dehydrogenase, which are essential enzymes in the TCA cycle (74). Similar to vitamins, dietary fatty acids influence B cell metabolism and the production of IgA (73, 75). A study showed that intestinal IgA production increased in mice maintained on a palmitic acid-enriched oil (76). Palmitoyl-CoA and serine act as substrates of serine palmitoyltransferase (SPT) and are subsequently converted into sphingolipids, such as ceramide, sphingosine, sphingomyelin, and S1P (77). Sphingolipids are a class of membrane lipids that also have biologic functions (78). For instance, an increase in ceramide concentrations may induce the proliferation of IgA PCs. Moreover, S1P can recruit IgA PCs into the intestine. These activation pathways may coincide with the changes in energy metabolism that occur in intestinal IgA PCs (76). Furthermore, metabolic intermediates of glycolysis, such as glucose monophosphate and fructose bis-phosphate, were detected preferentially in IgA PCs compared with naïve B cells, which implied that the shift to glycolysis-mediated energy metabolism likely is useful for the generation and production of IgA (73). For example, the glucose monophosphate and 3-phosphoglycerate generated through glycolysis are used in the pentose phosphate and serine biosynthetic pathways for nucleotide and amino acid synthesis, respectively (79). In addition, pyruvate, the metabolic product of glycolysis, can subsequently be converted to acetyl coenzyme A (acetyl-CoA) and used in fatty acid synthesis, which is required for B cell differentiation (80) (**Figure 3**).

In addition, dysbiosis and changes in microbiome diversity were recently shown to regulate the IgA–microbiota axis and influence multiple aspects of antitumor immunity by altering the risk of developing cancer and regulating responses to immunotherapy (81–83). It was shown that gut and lung microbiota themselves are capable of promoting IgA CSR through antigen presentation by CD103^+^ DCs and induction of IL-10 and TGF-β (84). Conversely, a decrease in intestinal IgA responses and structural immaturity of PPs occurred in germ-free mice (85). Gut commensal microbes can produce biologically active metabolites, represented by the short-chain fatty acids (SCFAs) acetate, propionate and butyrate, which are all reported to promote IgA CSR (86, 87). Recently, Isobe et al. discovered that commensal-bacteria-derived butyrate up-regulated the production of TGF-β and all-trans retinoic acid by CD103^+^CD11b^+^DCs, both of which promote the T-cell-independent IgA response in the colon (88) (**Figure 3**).

A possible role of IgA in tumor immunity arises from its dynamic relationship with diet and the microbiota. Hyper-nutrition and alcohol consumption not only regulate the magnitude of B cell activity and effector function but also change the levels and diversity of IgA, which may further enhance tumor development (14, 89, 90). In addition, Piper et al. identified that the aryl-hydrocarbon receptor (AhR), a ligand-activated transcription factor that senses dietary, microbial, and metabolic cues, binds to the IL-10 locus in murine cells (91). The supplementation with microbially derived SCFA butyrate supports Breg to produce IL-10 by amplifying AhR activation (91). IL-10 upregulates the expression of AID and triggers the induction of SHM and CSR from IgM to IgA, which may mediate tumor progression (20, 33).

## Intratumoral IgA Repertoires

RNA- and DNA-based analyses of Ig repertoires and intratumoral T-cell receptors have received considerable attention as powerful tools for identifying prognostic and predictive cancer biomarkers, such as clonality metrics (2, 92). In particular, assessment of the degree of clonality and the amount of somatic hypermutations in Ig gene segments can offer prognostic value for some types of cancer (93). Clonality reflects mainly the presence of plasma cell clonal expansion. TCGA RNA-seq data on melanoma samples have demonstrated that a high IgG1 proportion is strongly associated with the presence of large clonal expansions, contributing to a better prognosis. In contrast, a high IgA proportion correlates with low clonality, resulting in a negative outcome (2). These results can be explained by a focused immune response leading to the production of high-affinity, tumor-specific IgG1 antibodies; conversely, switching of B cells to the IgA isotype is not driven by particular antigens but is a passive consequence of the intratumoral suppressive cytokine environment (40). Therefore, it can be hypothesized that dual effect of IgA on tumor immunity is highly associated with clonal expansion of PCs. High IgA expression and high clonality, implying that IgA may be driven by a focused immune response and may result in a better prognosis, whereas high IgA expression and low clonality mean that an immunosuppressive microenvironment drives IgA production and is likely to contribute to poor outcomes in some specific malignancies.

## Conclusion

IgA and IgA^+^ cells are active participants that can fundamentally orchestrate the immune response in the TME. IgA can exert pro-tumorigenic roles in cancer by inducing the release of immunosuppressive cytokines, forming immune complexes with tumor or non-tumor antigens, and interacting with immunosuppressive cells, such as MDSCs or Tregs. However, IgA can also promote anticancer immunity and suppress tumor growth. These opposing effects can be explained by the influence of different tumor types, environmental factors, and host factors, which elicit a different antigen specificity of IgA, as well as the inconsistent interaction between IgA and tumor cells. Decisions on the application of IgA-targeted therapies and their combination with other therapies should be based on an advanced understanding of the prevailing nature of IgA involvement in tumor–immune interactions in each cancer subtype or even in each patient. Therefore, analysis of intratumoral IgA repertoires and determination of their metabolites are becoming powerful tools for identifying prognostic and predictive cancer biomarkers.

## Author Contributions

Conceptualization: ZZ, RL, and CM. Writing original draft: ZZ, KN, and MW. Supervision: YC, RL, and CM. All authors contributed to the article and approved the submitted version.

## Funding

This work was supported by the Shanghai Rising-Star Program (19QA1406900), the National Science Foundation of China (81873948, 82072213, 81972666, 81730045), and the National Science and Technology Major Project of China (2020YFC2008402).

## Conflict of Interest

The authors declare that the research was conducted in the absence of any commercial or financial relationships that could be construed as a potential conflict of interest.

## Publisher’s Note

All claims expressed in this article are solely those of the authors and do not necessarily represent those of their affiliated organizations, or those of the publisher, the editors and the reviewers. Any product that may be evaluated in this article, or claim that may be made by its manufacturer, is not guaranteed or endorsed by the publisher.
